# Changes in Subcellular Distribution of *n*-Octanoyl or *n*-Decanoyl Ghrelin in Ghrelin-Producing Cells

**DOI:** 10.3389/fendo.2013.00084

**Published:** 2013-07-09

**Authors:** Yoshihiro Nishi, Hiroharu Mifune, Akira Yabuki, Yuji Tajiri, Rumiko Hirata, Eiichiro Tanaka, Hiroshi Hosoda, Kenji Kangawa, Masayasu Kojima

**Affiliations:** ^1^Department of Physiology, School of Medicine, Kurume University, Kurume, Japan; ^2^Institute of Animal Experimentation, School of Medicine, Kurume University, Kurume, Fukuoka, Japan; ^3^Laboratory of Veterinary Clinical Pathology, Joint Faculty of Veterinary Medicine, Kagoshima University, Kagoshima, Japan; ^4^Division of Endocrinology and Metabolism, School of Medicine, Kurume University, Kurume, Fukuoka, Japan; ^5^Department of Pediatrics and Child Health, School of Medicine, Kurume University, Kurume, Fukuoka, Japan; ^6^Department of Biochemistry, National Cerebral and Cardiovascular Center Research Institute, Osaka, Japan; ^7^Molecular Genetics, Institute of Life Science, Kurume University, Kurume, Fukuoka, Japan

**Keywords:** decanoyl ghrelin, fasting, ghrelin *O*-acyltransferase, immunoelectron microscopy, medium-chain triglycerides, octanoyl ghrelin, radioimmunoassay, secretory granules

## Abstract

**Background:** The enzyme ghrelin *O*-acyltransferase (GOAT) catalyzes the acylation of ghrelin. The molecular form of GOAT, together with its reaction *in vitro*, has been reported previously. However, the subcellular processes governing the acylation of ghrelin remain to be elucidated.

**Methods:** Double immunoelectron microscopy was used to examine changes in the relative proportions of secretory granules containing *n*-octanoyl ghrelin (C8-ghrelin) or *n*-decanoyl ghrelin (C10-ghrelin) in ghrelin-producing cells of mouse stomachs. The dynamics of C8-type (possessing C8-ghrelin exclusively), C10-type (possessing C10-ghrelin only), and mixed-type secretory granules (possessing both C8- and C10-ghrelin) were investigated after fasting for 48 h or after 2 weeks feeding with chow containing glyceryl-tri-octanoate (C8-MCT) or glyceryl-tri-decanoate (C10-MCT). The dynamics of C8- or C10-ghrelin-immunoreactivity (ir-C8- or ir-C10-ghrelin) within the mixed-type granules were also investigated.

**Results:** Immunoelectron microscopic analysis revealed the co-existence of C8- and C10-ghrelin within the same secretory granules (mixed-type) in ghrelin-producing cells. Compared to control mice fed standard chow, the ratio of C10-type secretory granules increased significantly after ingestion of C10-MCT, whereas that of C8-type granules declined significantly under the same treatment. After ingestion of C8-MCT, the proportion of C8-type secretory granules increased significantly. Within the mixed-type granules the ratio of ir-C10-ghrelin increased significantly and that of ir-C8-ghrelin decreased significantly upon fasting.

**Conclusion:** These findings confirmed that C10-ghrelin, another acyl-form of active ghrelin, is stored within the same secretory granules as C8-ghrelin, and suggested that the types of medium-chain acyl-molecules surrounding and available to the ghrelin-GOAT system may affect the physiological processes of ghrelin acylation.

## Introduction

Ghrelin is an acylated peptide hormone mainly produced in the stomach (1). A main acyl-form of ghrelin in rodents and humans is an *n*-octanoyl ghrelin (C8-ghrelin), a serine 3 residue (Ser^3^) of which is modified by an *n*-octanoic acid (2, 3). The acylation of ghrelin is catalyzed by an enzyme: ghrelin *O*-acyltransferase (GOAT: previously known as MBOAT4) that belongs to the superfamily of membrane-bound *O*-acyltransferase (MBOAT) (4, 5). Since the discovery of GOAT (6, 7), many *in vitro* and *in vivo* studies on the mechanism of ghrelin acylation by this enzyme have been carried out (8–11). However, several discrepancies have emerged between the *in vitro* and *in vivo* findings (5, 12). For example, as regards the stomach content of acyl-ghrelins modified by a fatty acid with a carbon-chain shorter or a longer than eight (C8), we have detected a very low content of *n*-hexanoyl ghrelin (C6-ghrelin) in stomachs of mice under physiological conditions (i.e., without the ingestion of glyceryl-tri-hexanoate: a medium-chain triglyceride (MCTs) composed of three sets of *n*-hexanoyl group, C6-MCT) (13). We have also detected a considerable amount of *n*-decanoyl ghrelin (C10-ghrelin) in stomachs of mice, rabbits, or golden hamster fed *ad libitum* a standard chow (12). Furthermore, after fasting, the content of C10-ghrelin in mouse stomachs increased to nearly one third that of C8-ghrelin (14). These observations *in vivo* did not match the findings *in vitro* showing that GOAT has a preference for *n*-hexanoyl-CoA (C6-CoA) over *n*-octanoyl-CoA (C8-CoA), and also has greater preference for C8-CoA than *n*-decanoyl-CoA (C10-CoA) as an acyl donor (9).

Several groups have used immunoelectron microscopy to study the subcellular distribution of ghrelin (15–17) and ghrelin-related molecules (18, 19). However, there have been no reports concerning the distributional changes of acyl-ghrelins within ghrelin-producing cells (subcellular dynamics of acyl-ghrelins) under different nutritional conditions.

The present study used double immunoelectron microscopy to confirm the subcellular distribution of C8- and C10-ghrelins within ghrelin-producing cells. To shed light on the subcellular processes of ghrelin acylation, changes in the distribution of C8- or C10-ghrelin in ghrelin-producing cells were investigated after fasting or after ingestion of glyceryl-tri-octanoate (C8-MCT) or glyceryl-tri-decanoate (C10-MCT). In order to relate the present electron microscopic findings to the biochemical findings reported previously (11, 13, 14, 20), we also examined the change in stomach contents of C8- or C10-ghrelin by respective radioimmunoassay system (RIA), and studied the changes of ghrelin and GOAT mRNA levels in the mouse stomach.

## Materials and Methods

### Animals

Male C57BL/6J mice (Jcl: C57BL/6J, CLEA Japan, Inc., Osaka, Japan) weighing 20–25 g (10–13 weeks old) were used in this study. The animals were maintained under controlled temperature (24 ± 1°C), humidity (55 ± 5%), and light conditions (light on 07:00–19:00 h) with free access to standard laboratory chow (CE-2, CLEA Co. Ltd., Osaka, Japan) and water. Stomach and plasma samples from mice were obtained under anesthesia with sodium pentobarbital 30 mg/kg i.p. (Nembutal™, Dainippon Pharmaceutical Co., Ltd., Osaka, Japan). All experiments were undertaken in accordance with the *Guidelines for Animal Experimentation*, Kurume University.

### Schedule for the ingestion of medium-chain triglycerides

To examine the effect of dietary MCTs on the cellular distribution of stomach *n*-octanoyl ghrelin (C8-ghrelin) or *n*-decanoyl ghrelin (C10-ghrelin), mice (*n* = 5 in each ingestion group) were fed chow mixed with 3% (wt/wt) glyceryl-tri-octanoate or tri-decanoate (C8-MCT or C10-MCT; Wako Pure Chemical, Osaka, Japan) for 2 weeks as described previously (13, 21). The control animals were fed a CE-2 pellet diet and water *ad libitum*. Body weights of mice were measured before and after feeding with chow containing C8- or C10-MCT (C8-MCT-fed or C10-MCT-fed), and compared to those of control mice fed a standard laboratory chow *ad libitum* (Control). Daily food intake of the mouse (g/day/mouse) in each feeding condition (C8-MCT, C10-MCT, or Control) was also estimated by measuring the weight of chow every 24 h-period.

### Schedule for fasting experiment

Prior to performing the fasting experiment, the mice had free access to food and water. The fasting time was calculated from the time when food was withdrawn on the first day of the experiment. For the sampling from fasted mice (*n* = 5), food was withdrawn at 8:00 a.m. on the first day of the experiment and samples (stomach and plasma) were obtained at 8:00 a.m. on the third day (two-overnight) of the experiment. Body weights of mice before and after fasting were measured and compared.

### Immunohistochemistry for C8- and C10-ghrelin

The fundi of the stomach in the control mice (fed with free access to standard laboratory chow, *n* = 5), mice receiving chow with glyceryl-tri-octanoate (C8-MCT, *n* = 5) or tri-decanoate (C10-MCT, *n* = 5), and fasted mice (*n* = 5) were collected and fixed in Zamboni’s solution and routinely embedded in paraffin. Immunohistochemical staining was performed according to the modified avidin-biotin-peroxidase complex (ABC) technique described in our previous report (17). For C8- and C10-ghrelin immunohistochemical study, rabbit antiserum against C8-ghrelin diluted 1: 100,000 or rabbit antiserum against C10-ghrelin diluted 1:2000 was used as the primary antibody. Negative control studies were performed with anti-C8- or C10-ghrelin antisera, each of which had been abolished by 10 μg of synthesized C8-ghrelin or C10-ghrelin, respectively. Negative control studies were also done by omitting antisera against C8-ghrelin or C10-ghrelin. These negative controls showed no immunoreactions. For light-microscopic morphometry, three to five sections from each mouse stomach (*n* = 5 mice in each group) were observed at random using an ocular micrometer, and the number of immunopositive cells for C8- or C10-ghrelin per unit area of glandular portions (mm^2^) was counted.

### Double immunofluorescence for C8- and C10-ghrelin

Immunofluorescent staining was performed according to the double immunofluorescence technique described in our previous report (17). For C8- and C10-ghrelin immunofluorescent study, mouse monoclonal antibody against the N-terminal sequence of C8-ghrelin (Mitsubishi Kagaku Iatron Inc., Tokyo, Japan) diluted 1:2000 and rabbit antiserum against C10-ghrelin diluted 1:3000 were used as the primary antibody, respectively. Three sections from each mouse in control group were observed.

### Immunoelectron microscopy for C8- or C10-ghrelin

Samples for immunoelectron microscopy were prepared as described previously (17). Ultrathin sections were labeled by the post-embedding double immunogold labeling method as described previously (22) with slight modification. The double immunogold labeling was carried out using the two polyclonal antibodies against C8-ghrelin and C10-ghrelin. One face of a section was incubated in rabbit anti-C8-ghrelin antibody diluted 1:2000, and anti-rabbit IgG (British Biocell International, Cardiff, UK) conjugated with 20 nm gold particles (large particles) was used for the immunogold labeling of this face of the section. Rabbit anti-C10-ghrelin antibody (diluted 1:400) and anti-rabbit IgG (British Biocell International) conjugated with 10 nm gold particles (small particles) were used for the immunogold labeling of the other face of the section. For morphometric analysis, at least 10 electron micrographs of ghrelin cells were taken from each animal at a primary magnification of ×20,000 and printed at a final magnification of ×50,000.

### Quantitative immunoelectron microscopic analyses for C8- and C10-ghrelin

Immunogold ultrastructural morphometric analysis was done as described in the previous reports (16, 23, 24). Approximately three to five photographs of ghrelin-producing cells per section of fundi were randomly selected from three to five sections per mouse in the stomach of each mouse (over 100 images). Based on our previous findings for the average diameter of ghrelin-positive granules in mice (277 ± 11.1 nm) (17), we randomly chose secretory granules of 250–300 nm diameter from one ghrelin-producing cell per photograph. The observed counts for immunogold-particles within a single secretory granule were used to construct numerical and percentage frequency distributions for C8- or C10-ghrelin in the control, C8-MCT-fed, C10-MCT-fed, and fasted mice (*n* = 5 mice in each group). Immunogold-labeled secretory granules containing only immunoreactivity for C8-ghrelin (large immunogold-particles) were defined as C8-type; those showing immunoreactivity only for C10-ghrelin (small immunogold particles) were defined as C10-type; and those containing immunoreactivity for both C8- and C10-ghrelin (both large and small immunogold particles) were mixed-type. The number of secretory granules (C8-, C10-, or mixed-type) in ghrelin-producing cells of C8-MCT-fed, C10-MCT-fed, or fasted mice was counted (approximately 250 secretory granules per each group of mice), and the proportion (%) of respective granule type was calculated within ghrelin-producing cells from each group of mice (C8-MCT-fed, C10-MCT-fed, fasted, or fed *ad libitum*; *n* = 5 mice in each group). Furthermore, with regard to the mixed-type secretory granules that were immunopositive for both C8- and C10-ghrelin, the number of small immunogold particles (reflecting the C8-ghrelin-immunoreactivity) and large immunogold particles (reflecting the C10-ghrelin-immunoreactivity) within a single secretory granule were counted separately, and the proportions of each type were calculated relative to the total number (small plus large particles). The average rate of immunoreactivity for C8- or C10-ghrelin within the mixed-type secretory granules was calculated from three to five photographs of double immunoelectron microscopy per mouse. Thereafter, the changes in the proportions of C8- or C10-ghrelin-immunoreactivity per mixed-type secretory granule were evaluated under fasting conditions and compared with the results in control mice fed standard chow *ad libitum* (*n* = 5 mice in each group).

### RIAs for C8-ghrelin, C10-ghrelin, and total ghrelin

The RIA for C8- or C10-ghrelin (C8-ghrelin RIA, C10-ghrelin RIA) was performed as described previously for rat and mouse ghrelin (14, 25, 26). The anti-C8-ghrelin antiserum exhibited 100% cross-reactivity with rat, mouse, and human C8-ghrelin but does not recognize *des*-acyl-ghrelin. The anti-C10-ghrelin antiserum exhibited 100% cross-reactivity with rat, mouse, and human C10-ghrelin but does not recognize *des*-acyl-ghrelin. The cross-reactivity of anti-C10-ghrelin antiserum against C8-ghrelin was less than 2%. Cross-reactivity of both anti-C8- and anti-C10-ghrelin antisera to *n*-butyryl, *n*-hexanoyl, *n*-lauryl, and *n*-palmitoyl ghrelin was all less than 5%. The RIA for total ghrelin was also performed as described previously for rat and mouse ghrelin (14, 25). The antiserum used for the total ghrelin RIA recognized all ghrelin peptides with intact C-terminal sequences irrespective of their N-terminal acylation, and exhibited complete cross-reactivity with human, mouse, and rat forms of ghrelin. Stomach or plasma samples for C8-ghrelin, C10-ghrelin, or total ghrelin RIA from mice were prepared as described previously (14, 25, 27).

### Reverse transcriptase polymerase chain reaction for mRNAs of ghrelin and GOAT

The expression levels of mRNA for ghrelin and GOAT in stomachs of mice were examined using semi-quantitative reverse transcriptase polymerase chain reaction (PCR) as described previously (28, 29). The PCR was performed using a commercially available PCR kit (Go-Taq Master Mix; Promega, Madison, WI, USA) with each primer set necessary to amplify the transcripts for ghrelin (Accession No. NM_021488.4, 32 cycles, 329 bps), GOAT (Accession No. NM_001126314, 36 cycles, 141 bps), and β-actin (Accession No. XM_136101, 22 cycles, 224 bps). The sense or reverse primer for the amplification of mouse ghrelin mRNA was 5′-AGTCCTGTCAGTG GTTACTTG-3′ or 5′-AGGCCTGTCCGTGGTTACTTG-3′, respectively. The sense or reverse primer for the amplification of mouse GOAT mRNA was 5′-GGGCCAGGTACCTCTTTCTC-3′ or 5′-GCCTATGGACTTCCTGTGGA-3′, respectively. The sense or reverse primer for the amplification of mouse β-actin mRNA was 5′-CCTAGCACCATGAAGATCAA-3′ or 5′-TTTCTGCACAAGTTAGG TTTTGTCAA-3′, respectively. The NIH-Image program was used to determine the relative amount of each PCR product after gel electrophoresis, and the amount was normalized using simultaneously amplified β-actin (30).

### Statistical analysis

Data were presented as the means ± SD. The statistical significance was determined by ANOVA, two-tailed Student’s paired *t*-test, or Chi-square test. A *p*-value < 0.05 was considered to be statistically significant on ANOVA and Student’s *t*-test. A *p*-value < 0.016 was considered to be statistically significant concerning the difference among the proportion for types of secretory granules (Chi-square test followed by Bonferroni’s procedure for multiple tests of significance). All tests were performed using SAS version 9.2 (SAS Institute, Cary, NC, USA).

## Results

### Body weights and daily food intake of mice

Body weight of mice before the treatment with MCT-containing chow was 24.5 ± 1.2 g in the control group fed a standard chow (CE-2, CREA, Japan), 24.2 ± 0.7 g in the C8-MCT-fed group, and 23.4 ± 0.6 g in the C10-MCT-fed group (*n* = 5 in each group). After 2 weeks of the respective feeding regimens, body weights of mice fed control chow, C8-MCT- and C10-MCT-containing chow were 25.2 ± 1.7, 25.2 ± 0.9, and 24.0 ± 0.2 g, respectively. Among the three groups of mice, there were no significant differences in body weights before or after the treatment. The average daily food consumption (g/day) in each group was 3.36 ± 0.49 g for control group, 3.18 ± 0.44 g for C8-MCT-fed group, and 3.06 ± 0.40 g for C10-MCT-fed group. Again, there were no significant differences in daily food consumption among these three groups. Average body weights of mice upon fasting for 48 h (16.7 ± 1.8 g, *n* = 5) were significantly (*p* < 0.05) lower than that of control mice fed *ad libitum* a standard chow.

### Immunoreactivity for C8- or C10-ghrelin in stomachs of mice

**Figure 1:**
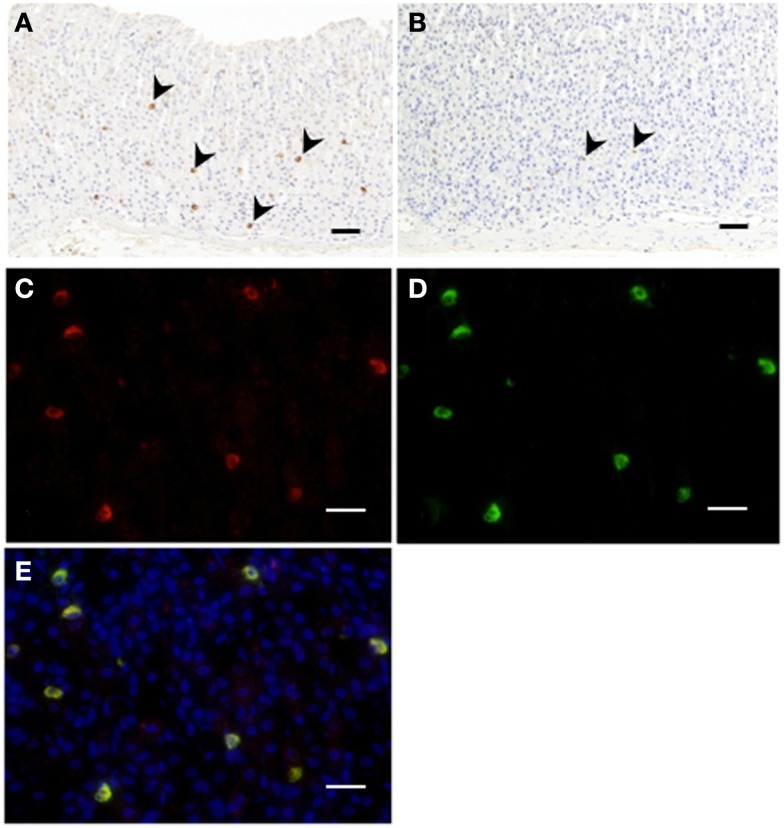
**Immunohistochemistry for C8-ghrelin (A) or C10-ghrelin (B) in fundi of stomachs in control mice**. The density of DAB staining in cells reflecting C10-ghrelin-immunoreactivity (ir-C10-ghrelin) was lower than that of ir-C8-ghrelin **(A,B)**. Immunofluorescence in fundi of stomachs in control mice **(C–E)**. Ir-C8-ghrelin **(C)** labeled with Alexa 555 (red), and ir-C10-ghrelin **(D)** labeled with Alexa 488 (green) are observed in the same section. The yellow color in the merged image **(E)** indicated a co-localization of ir-C8- and ir-C10-ghrelin. Arrowheads indicate ghrelin-positive cells **(A,B)**. Scale bars represent 100 μm **(A,B)** and represent 25 μm **(C–E)**.

Immunopositive cells for C8-ghrelin (ip-C8-ghrelin) and C10-ghrelin (ip-C10-ghrelin) in stomachs of control mice given free access to standard laboratory chow, were sparsely distributed in the middle to lower part of the gastric mucosal layer, where they were moderately abundant (Figures 1A,B). A small amount of immunopositive cells for C8- or C10-ghrelin was also detected in the gastric submucosal layer. No immunopositive cells for C8- or C10-ghrelin were observed in the gastric superficial epithelial layer. In control mice, the density of DAB staining, reflecting the C10-ghrelin-immunoreactivity (ir-C10-ghrelin), within the ip-C10-ghrelin cells was lower than that for the C8-ghrelin-immunoreactivity (ir-C8-ghrelin) within the C8-ghrelin-immunopositive (ip-C8-ghrelin) cells (Figures 1A,B). In the fundic mucosa of the mouse stomach, the density of ip-C10-ghrelin cells (21.3 ± 3.7 cells/mm^2^ mucosa) was approximately one third that of ip-C8-ghrelin cells (57.5 ± 4.6 cells/mm^2^ mucosa) in the same section (Table 1).

**Table 1:** **Numbers of immunopositive cells for C8- or C10-ghrelin in stomachs of mice**.

	ip-C8-ghrelin	ip-C10-ghrelin
C8-MCT-fed	140.6 ± 15.5**^,$^	18.7 ± 3.1^#,$^
C10-MCT-fed	51.4 ± 1.9*	96.5 ± 15.4**^,#^
Fasted	51.3 ± 3.7	47.7 ± 4.1**
Control	57.5 ± 4.6	21.3 ± 3.7

Data represent mean ± SD for the number of immunopositive cells for C8-ghrelin (ip-C8-ghrelin) or C10-ghrelin (ip-C10-ghrelin) per unit area (mm^2^) of the stomach mucosa (the average value obtained from three to five sections per each stomach) (n = 5 mice in each group). C8-MCT-fed, stomachs of mice fed with glyceryl-tri-octanoate containing chow for 2 weeks; C10-MCT-fed, those fed with glyceryl-tri-decanoate containing chow for 2 weeks; Fasted, those fasted for 48 h with free access to water; Control, those fed ad libitum with standard laboratory chow (CE-2). *p < 0.05; **p < 0.01 vs. values in Control; ^$^p < 0.01 vs. values in C10-MCT-fed; ^#^p < 0.01 vs. values in Fasted.

### Immunofluorescence of C8- and C10-ghrelin in stomachs of mice

Double immunostaining for ir-C8-ghrelin and ir-C10-ghrelin revealed a co-localization of the immunoreactivity for both C8-ghrelin (red fluorescence, Figure 1C) and C10-ghrelin (green fluorescence, Figure 1D) within the same cells (yellow fluorescence in the merged image, Figure 1E) in mouse stomachs fed standard laboratory chow *ad libitum*. These findings clearly indicated the co-localization of C8- and C10-ghrelin in the same cell population.

### Intracellular distribution of C8- or C10-ghrelin-immunoreactivity within the ghrelin-producing cells

**Figure 2:**
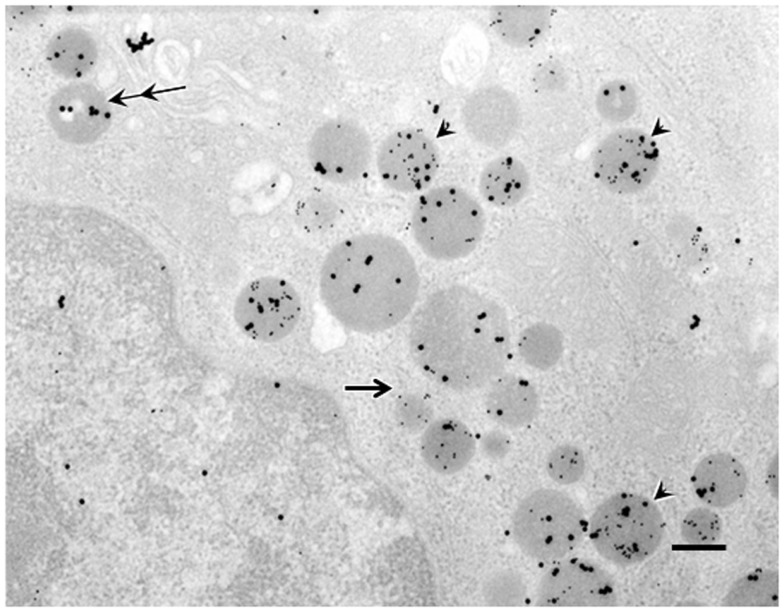
**Double immunogold labeling in ghrelin-producing cells in the stomachs of control mice**. Large particles of immunogold (20 nm in diameter) or small particles of immunogold (10 nm in diameter) demonstrated immunoreactivity for C8-ghrelin (ir-C8-ghrelin) or C10-ghrelin (ir-C10-ghrelin), respectively. Over 70% of secretory granules in ghrelin-producing cells exhibited both ir-C8-ghrelin and ir-C10-ghrelin (arrowheads). Occasionally (10–20% of the secretory granules), there appeared granules stained only for ir-C8-ghrelin reflected by large particles (double arrows) or ir-C10-ghrelin reflected by small particles of immunogold (single arrow) in ghrelin-producing cells. Scale bar represents 200 nm.

Double immunogold labeling of C8-ghrelin and C10-ghrelin revealed that, in control mice, over 70% of the secretory granules in ghrelin-producing cells possessed both large (20 nm in diameter) and small (10 nm in diameter) particles of immunogold (Figure 2). Two other types of secretory granules were also observed in these cells, one containing only the large particles of immunogold (reflecting *ir*-C8-ghrelin), and the other containing only the small particles of immunogold (reflecting ir-C10-ghrelin). We defined these three types of secretory granules as C8-type (possessing only the ir-C8-ghrelin), C10-type (possessing only the ir-C10-ghrelin), or mixed-type (possessing both ir-C8 and ir-C10-ghrelin). Aside from the secretory granules in ghrelin-producing cells, we observed an extremely small population of Golgi-complexes that were immunopositive for C8- and/or C10-ghrelin (Figure 2).

### The proportion of C8-type, C10-type, or mixed-type secretory granules within ghrelin-producing cells

**Figure 3:**
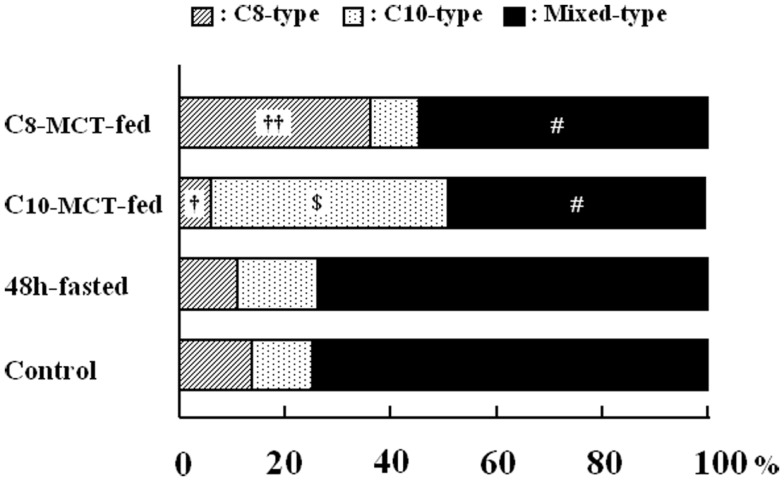
**The proportion of secretory granules that possessed immunoreactivity exclusively for C8-ghrelin (C8-type, cross-hatched column), or exclusively for C10-ghrelin (C10-type, dotted column), or for both C8- and C10-ghrelin (mixed-type, filled column) in ghrelin-producing cells of the stomach in control mice fed *ad libitum* a standard chow (Control), in mice fasted for 48 h (fasted), or in mice after 2-weeks ingestion of glyceryl-tri-octanoate (C8-MCT-fed) or glyceryl-tri-decanoate (C10-MCT-fed)**. Data were extracted and analyzed from approximately 250 secretory granules of ghrelin-producing cells in each group (*n* = 5 mice in each group). ^†^*p* < 0.01; ^††^*p* < 0.001 vs. control and fasted. *p* < 0.001 vs. control, C8-MCT-fed, and fasted. ^#^*p* < 0.001 vs. control and fasted.

As shown in Figure 3, we examined the intracellular proportions of the three types (C8-, C10-, mixed-type) of immunogold-labeled granules in ghrelin-producing cells under different nutritional conditions: fasted, C8-MCT-fed and C10-MCT-fed, and compared them with that of control mice fed *ad libitum*. Ghrelin-producing cells of control mice contained relatively small proportions of secretory granules that were exclusively immunopositive for either C8- or C10-ghrelin (C8-type; 13.8 ± 3.3%, C10-type; 9.2 ± 3.5%), whereas approximately 80% (77.1 ± 6.4%) of the secretory granules were mixed-type. In mice fed for 2 weeks with chow containing 3% glyceryl-tri-octanoate (C8-MCT-fed), the ratio of C8-type secretory granules (38.8 ± 5.4%) to the total number of ghrelin-secretory granules (the sum of C8-, C10-, and mixed-type) increased significantly (*p* < 0.01) compared with the control (13.8 ± 3.3%), while that of C10-type granules (6.8 ± 2.1%) in C8-MCT-fed mice slightly decreased in comparison to the control mice (9.2 ± 3.5%). The ratio of mixed-type secretory granules in ghrelin-producing cells of C8-MCT-fed mice (54.4 ± 5.9%) also decreased significantly (*p* < 0.001) compared with control mice (77.1 ± 6.4%). When we fed mice with chow containing 3% glyceryl-tri-decanoate for 2 weeks (C10-MCT-fed), the ratio of C10-type secretory granules (47.2 ± 6.9%) increased significantly (*p* < 0.001) compared with the control (9.2 ± 3.5%), while that of C8-type granules (4.6 ± 1.3%) in C10-MCT-fed mice decreased significantly (*p* < 0.001) compared with the control mice (13.8 ± 3.3%). The ratio of mixed-type secretory granules in ghrelin-producing cells of C10-MCT-fed mice (48.1 ± 6.3%) also decreased significantly (*p* < 0.001) relative to control mice (77.1 ± 6.4%). In contrast, after fasting, there were no significant changes in the proportions of these three types of secretory granules (C8-type, 10.5 ± 2.9%; C10-type, 15.3 ± 3.3%; mixed-type, 74.2 ± 5.3%) in comparison to those in control mice.

### The effect of fasting on the proportion of C8- or C10-ghrelin-immunoreactivity within the mixed-type secretory granules

In control mice (fed *ad libitum* a standard chow), the proportions of C8-ghrelin-immunoreactivity (ir-C8-ghrelin) and C10-ghrelin-immunoreactivity (ir-C10-ghrelin) within the mixed-type granules (as determined by the proportion of large or small particles of immunogold within the secretory granule) were 46.5 ± 3.0 and 53.5 ± 3.1%, respectively. After fasting for 48 h, the proportion of ir-C8-ghrelin within the mixed-type secretory granules (reflected by the number of large particles of immunogold) fell to 33.7 ± 2.1% of the total immunoreactivity of ghrelin (the sum of ir-C8- and ir-C10-ghrelin reflected by the number of large and small immunogold particles). Upon fasting, the proportion of ir-C10-ghrelin within the mixed-type secretory granules, which was reflected by the number of small particles of immunogold, increased to 66.2 ± 1.9% of total ghrelin immunoreactivity.

When we defined the average rate of ir-C8-ghrelin within the mixed-type secretory granules in one of the control mice as 1.0, and compared the relative value for the proportion of ir-C8-ghrelin before and after fasting, the value for the rate of ir-C8-ghrelin in fasted mice (0.76 ± 0.06) was significantly lower (*p* < 0.001) than that in control mice (1.05 ± 0.07) (*n* = 5 mice in each group). In contrast, the relative value for the proportion of ir-C10-ghrelin in fasted mice (1.19 ± 0.05) was significantly higher (*p* < 0.001) than that in control mice (0.96 ± 0.05) (*n* = 5 mice in each group).

### Contents of C8- or C10-ghrelin-immunoreactivity in stomachs of mice

As shown in Table 2, the stomach content of C8-ghrelin, as measured by C8-ghrelin RIA, was significantly larger (*p* < 0.01) in C8-MCT-fed stomachs than that in control stomachs. The stomach content of C10-ghrelin, which was measured by C10-ghrelin RIA, was significantly smaller (*p* < 0.05) in C8-MCT-fed stomachs, and was significantly larger (*p* < 0.01) in C10-MCT-fed stomachs compared with the controls. The stomach content of total ghrelin (measured by total ghrelin RIA which recognized both acyl- and *des*-acyl-ghrelin with intact C-termini) in both C8-MCT-fed and C10-MCT-fed stomachs were slightly but significantly decreased (*p* < 0.05) compared to that of control stomachs. Upon fasting, stomach contents of both C8-ghrelin and total ghrelin declined significantly (*p* < 0.05), and that of C10-ghrelin increased significantly (*p* < 0.01) than that in stomachs of control mice.

**Table 2:** **Stomach contents of C8- or C10-ghrelin under different nutritional conditions**.

	C8-ghrelin	C10-ghrelin	Total ghrelin
C8-MCT-fed	3.62 ± 0.33**^,#,$^	0.13 ± 0.02*^,#,$^	6.57 ± 0.79*
C10-MCT-fed	2.70 ± 0.36^$^	1.21 ± 0.12**^,$^	6.49 ± 0.80*
Fasted	1.63 ± 0.32*	0.58 ± 0.10**	6.57 ± 0.71*
Control	2.29 ± 0.23	0.27 ± 0.20	7.61 ± 0.66

Data represent mean ± SD for the stomach contents (pmol/mg-tissue) of each ghrelin molecule (C8-ghrelin, C10-ghrelin, or total ghrelin) measured by C8-ghrelin RIA, C10-ghrelin RIA, or total ghrelin RIA (n = 5 mice in each group). Control, fed ad libitum a control laboratory chow (the same group of mice used for the electron- and light-microscopic analysis); C8-MCT-fed, fed with chow containing glyceryl-tri-octanoate; C10-MCT-fed, fed with chow containing glyceryl-tri-decanoate; Fasted, fasted for 48 h with free access to water. *p < 0.05; **p < 0.01 vs. values in Control. ^#^p < 0.01 vs. values in C10-MCT-fed. ^$^p < 0.01 vs. values in Fasted.

### Expression levels of mRNAs for ghrelin or GOAT in stomachs of mice

The relative expression levels of mRNA for ghrelin, corrected by β-actin levels, in stomachs of fasted mice were significantly higher (*p* < 0.01) than in stomachs of control mice fed *ad libitum* a standard laboratory chow (Table 3). However, the levels of ghrelin mRNA in stomachs of C8-MCT or C10-MCT-fed mice did not differ from that in control mice. The relative expression levels of GOAT mRNA in fasted or C8-MCT-fed stomachs of mice did not differ from that in control stomachs. Whereas, the levels of GOAT mRNA in stomachs of C10-MCT-fed mice were significantly (*p* < 0.05) lower than those in control and C8-MCT-fed mice.

**Table 3:** **Expression levels of ghrelin and GOAT mRNAs in stomachs of mice under different nutritional conditions**.

	Ghrelin	GOAT
C8-MCT-fed	101.2 ± 13.6	105.4 ± 9.0
C10-MCT-fed	93.0 ± 8.7	82.8 ± 9.2*^,$^
Fasted	174.0 ± 0.33**	103.6 ± 18.5
Control	100.0 ± 7.8	100.0 ± 11.4

Data represent mean ± SD for the relative expression level of mRNAs for ghrelin or ghrelin O-acyltransferase (GOAT) measured by semi-quantitative RT-PCRs (n = 5 mice in each group). C8-MCT-fed, fed with glyceryl-tri-octanoate containing chow; C10- MCT-fed, fed with glyceryl-tri-decanoate containing chow; Fasted, fasted for 48 h with free access to water; Control, fed ad libitum with standard laboratory chow (the same group of mice used for the electron- and light-microscopic analysis). *p < 0.05; **p < 0.01 vs. values in Control. ^$^p < 0.05 vs. values in C8-MCT-fed. Expression level for the respective mRNAs for ghrelin, or GOAT in one of the control mice was defined as 100.

### Plasma concentrations of C8- or C10-ghrelin-immunoreactivity in mice

As shown in Table 4, plasma levels of C8-ghrelin increased significantly after fasting for 48 h. In contrast, plasma levels of C8-ghrelin did not change after the ingestion of C8-MCT for 2 weeks. Similarly, the change in C10-ghrelin level after ingestion of C10-MCT was far smaller than that seen upon fasting.

**Table 4:** **Plasma ghrelin levels under different nutritional conditions**.

	C8-ghrelin	C10-ghrelin	Total ghrelin
C8-MCT-fed	25.0 ± 5.5^#^	11.4 ± 2.0^##^	298.4 ± 56.2
C10-MCT-fed	21.0 ± 5.7^#^	13.8 ± 1.3^##^	351.8 ± 66.4
Fasted	88.3 ± 39.8*	45.8 ± 23.9**	383.1 ± 231.9
Control	21.8 ± 7.0	9.7 ± 2.4	330.6 ± 132.5

Data represent mean ± SD for the plasma concentrations (fmol/ml) of each ghrelin molecule (C8-ghrelin, C10-ghrelin, or total ghrelin) measured by C8-ghrelin RIA, C10-ghrelin RIA, or total ghrelin RIA (n = 5 mice in each group). C8-MCT-fed, fed with glyceryl-tri-octanoate containing chow; C10-MCT-fed, fed with glyceryl-tri-decanoate containing chow; Fasted, plasma samples from mice upon fasting for 48 h; Control, control mice fed ad libitum a standard laboratory chow. *p< 0.05; **p < 0.01 vs. values in Control. ^#^p < 0.05; ^##^p< 0.01 vs. values in Fasted.

## Discussion

The present study by double immunoelectron microscopy confirmed our previous findings by light microscopy concerning the co-existence of C8- and C10-ghrelin-immunoreactivity (ir-C8- and ir-C10-ghrelin) within the same ghrelin-producing cells (14). On immunoelectron microscopy, we observed both ir-C8- and ir-C10-ghrelin within round and compact dense granules of X/A-like cell type, a characteristics of ghrelin-producing cells in rats (15), mice (18), and hamster (17).

Concerning the subcellular distribution of ir-C8- or ir-C10-ghrelin outside of secretory granules, we did not detect any significant signals of immunogold within the endoplasmic reticulum or Golgi complex. However, these findings did not disprove the putative concept (31) that the acylation of ghrelin by GOAT precedes the protease cleavage of pro-ghrelin to ghrelin, because the antibodies we used in this study possessed little or no cross-reactivity to pro-ghrelin peptides irrespective of their acylation status (12, 25).

After feeding mice with chow containing C8-MCT or C10-MCT for 2 weeks, a significant increase was noted in the proportion of C8- or C10-type secretory granules, respectively, to the total number of ghrelin-secretory granules in ghrelin-producing cells, while the proportion of mixed-type granules to the total number of ghrelin-secretory granules in ghrelin-producing cells declined significantly. These findings suggested that a certain proportion (10–20%) of the mixed-type secretory granules changed to C8-type or C10-type after a feeding regimen containing C8-MCT or C10-MCT, respectively. These findings also implied that the type of medium-chain acyl-molecules (i.e., C8-CoA or C10-CoA) surrounding the ghrelin-GOAT system has strong effect on the type of acyl-ghrelins (i.e., C8-ghrelin or C10-ghrelin) stored within the ghrelin-secretory granules.

When we evaluated the stomach contents of ir-C8- or ir-C10-ghrelin by RIA, the levels of ir-C10-ghrelin increased significantly in both C10-MCT-fed and fasted conditions, which was in line with previous reports (11–14, 21). In contrast, upon fasting, the ratios of secretory granules (C8-, C10-, or mixed-type) to the total number of ghrelin-secretory granules in ghrelin-producing cells did not differ from those in control mice fed *ad libitum*. However, in the same ghrelin-producing cells of fasted mice, we detected a significant increase in the proportion of ir-C10-ghrelin and a significant decline in the proportion of ir-C8-ghrelin within the mixed-type granules. On the other hand, we could not detect any significant change in the proportions of ir-C8- or ir-C10-ghrelin within the mixed-type granules after feeding mice with C8- or C10-MCT-containing chow for 2 weeks (data not shown here). These differences in the subcellular distribution of ir-C8- or ir-C10-ghrelin together with its kinetics under different nutritional conditions, such as fasting or constant feeding with C8- or C10-MCT, might offer important clues on the subcellular process of ghrelin acylation.

Two-weeks treatment with C8- or C10-MCT did not alter the expression levels of ghrelin mRNA in mouse stomachs, which was supported by our previous report (13). As for GOAT mRNA, no significant changes were detected except for a slight but significant decline after 2-weeks treatment with C10-MCT. Although the precise mechanism of suppression of GOAT mRNA level by C10-MCT-feeding remains to be solved, the influence of ghrelin and/or GOAT mRNA levels on the subcellular kinetics of ir-C8- or ir-C10-ghrelins appeared to be limited.

Differences in the dynamics of plasma ghrelin levels upon different nutritional conditions examined (i.e., upon fasting or 2 weeks feeding with MCT-containing chow), which may reflect the release of ghrelin from secretory granules into circulation, should also be considered to be one of the key processes governing the changes of ir-C8- or ir-C10-ghrelin within the secretory granule. In fact, changes in the level of C8- or C10-ghrelin in plasma upon fasting were far larger than those seen after feeding mice with chow containing C8-MCT or C10-MCT.

Based on the above findings, especially on those revealed by our double immunoelectron microscopy, one may speculate that the mechanism underlining ghrelin acylation is complicated, and cannot be explained simply by the enzymatic activity of GOAT within ghrelin-producing cells. However, our immunoelectron microscopic study on ghrelin acylation also has several limitations. Within the mixed-type secretory granules, relatively larger proportion of ir-C10-ghrelin was observed as compared with ir-C8-ghrelin. This finding, however, did not reflect the absolute content of ir-C8-, or ir-C10-ghrelin in the mixed-type granule, since we used different antibodies possessing different affinities to the C8- or C10-forms of acyl-ghrelin. In our study, we only compared the subcellular distribution of ir-C10- or ir-C8-ghrelin and changes in this distribution under different nutritional conditions, and did not look at the distribution or the changes of other acyl-ghrelins including *n*-hexanoyl ghrelin, an intriguing molecule whose production rate catalyzed by GOAT *in vitro* is far higher than that of C10-ghrelin (9, 10), while it’s biological activity is far smaller than that of C10- or C8-ghrelin (21, 32).

In conclusion, in this study we investigated changes in the subcellular distribution of ir-C8- or C10-ghrelin under different nutritional conditions by double immunoelectron microscopy. Present findings indicated that there existed several steps for the synthesis of acyl-ghrelins within ghrelin-secretory granules, and also implied that the types of medium-chain acyl-molecules surrounding the ghrelin-GOAT system affect the acylation process of ghrelin. Further study using immunoelectron microscopy on the subcellular distribution of acyl-ghrelins will shed light on the mechanism underlining the acylation process of ghrelin.

## Conflict of Interest Statement

The authors declare that the research was conducted in the absence of any commercial or financial relationships that could be construed as a potential conflict of interest.
